# Contact transmission of influenza virus between ferrets imposes a looser bottleneck than respiratory droplet transmission allowing propagation of antiviral resistance

**DOI:** 10.1038/srep29793

**Published:** 2016-07-19

**Authors:** Rebecca Frise, Konrad Bradley, Neeltje van Doremalen, Monica Galiano, Ruth A. Elderfield, Peter Stilwell, Jonathan W. Ashcroft, Mirian Fernandez-Alonso, Shahjahan Miah, Angie Lackenby, Kim L. Roberts, Christl A. Donnelly, Wendy S. Barclay

**Affiliations:** 1 Imperial College London, Faculty of Medicine, Division of Infectious Disease, Norfolk Place, London, W2 1PG, United Kingdom; 2 Public Health England, Colindale, London, United Kingdom; 3 MRC Centre for Outbreak Analysis and Modelling, Department of Infectious Disease Epidemiology, School of Public Health, Faculty of Medicine, Imperial College London, United Kingdom

## Abstract

Influenza viruses cause annual seasonal epidemics and occasional pandemics. It is important to elucidate the stringency of bottlenecks during transmission to shed light on mechanisms that underlie the evolution and propagation of antigenic drift, host range switching or drug resistance. The virus spreads between people by different routes, including through the air in droplets and aerosols, and by direct contact. By housing ferrets under different conditions, it is possible to mimic various routes of transmission. Here, we inoculated donor animals with a mixture of two viruses whose genomes differed by one or two reverse engineered synonymous mutations, and measured the transmission of the mixture to exposed sentinel animals. Transmission through the air imposed a tight bottleneck since most recipient animals became infected by only one virus. In contrast, a direct contact transmission chain propagated a mixture of viruses suggesting the dose transferred by this route was higher. From animals with a mixed infection of viruses that were resistant and sensitive to the antiviral drug oseltamivir, resistance was propagated through contact transmission but not by air. These data imply that transmission events with a looser bottleneck can propagate minority variants and may be an important route for influenza evolution.

Influenza viruses cause respiratory infections in annual seasonal outbreaks and intermittent pandemics. The spread of virus is difficult to control because transmission is efficient and may occur from asymptomatic or presymptomatic individuals^1^,^2^. Influenza is believed to spread through direct contact (DC) with contaminated persons or surfaces, as well as through the air in aerosols and respiratory droplets (RD) although the importance of these routes of transmission for spread of human influenza remains a subject of debate^3^,^4^. Some evidence suggests that the route of infection may influence the severity of outcome, with more severe disease resulting from RD acquisition, perhaps because virus in aerosols can reach the lower lung^5^,^6^. Based on this assumption, 50% or fewer of transmission events were estimated to occur by RD during the 2009 H1N1 pandemic^7^.

The ferret is the favoured animal model for studying influenza transmission. This is because ferrets and humans show a similar distribution of sialic acid (SA) receptors that enable incoming virus to enter target cells, and ferrets display clinical signs after infection reminiscent of influenza-like symptoms in humans^8^. In particular, ferrets have been utilized to predict the ability of a particular strain of animal influenza virus to transmit between humans (http://www.cdc.gov/flu/pandemic-resources/tools/risk-assessment.htm)^9^. This is appropriate because several of the barriers that restrict avian influenza viruses in humans also operate in ferrets. For example, both species have a paucity of α2,3 linked (SA) receptors, the preferred receptors for avian influenza viruses, in the respiratory tract^10^,^11^. Transmission of virus between ferrets also requires viruses to bind α2,6SA, have increased stability and efficient replication, all features of human-adapted influenza viruses^12^,^13^. Indeed, experimental data obtained in the ferret transmission model generally correlate with observations made in humans^14^. For example, the avian influenza virus H5N1 has rarely transmitted between humans, and then only under close contact conditions in households^15^. This virus does not transmit between ferrets even when animals are housed together^16^. Many swine influenza viruses transmit between ferrets in the same cage (DC) but do not pass through the air to ferrets in adjacent cages (RD)^17^,^18^. Since most zoonotic transmissions of swine influenza viruses have not developed to larger human outbreaks, the experimental findings imply that the ability of a newly emerged influenza virus to transmit by RD between ferrets would be an indicator of its ability to cause a human pandemic.

These observations suggest that RD transmission may exert a more stringent bottleneck than DC transmission. Implications for virus evolution are that whilst genetic diversity might expand in an infected host, only a fraction of that diversity might be transmitted. Comprehending the circumstances under which minority populations might be transmitted onwards is crucial for understanding the emergence of new traits such as drug resistance, antigenic drift or extended host range^19^,^20^,^21^.

To address the stringency of the influenza transmission bottleneck, several groups have analysed sequence variation between viruses in donor and recipient animals during transmission experiments. During the rather inefficient RD transmission of avian-like H5N1 or H1N1 viruses between ferrets, next generation sequencing (NGS) demonstrated that HA variants that had been minority genotypes in donor animals were uniquely transmitted, suggesting a tight bottleneck^22^,^23^. Indeed, Zaraket *et al*. also described a tight bottleneck that restricted the RD transmission of H7N9 avian influenza viruses to mammals^24^. In transmission experiments involving a fully transmissible human-adapted virus carrying barcodes to trace populations Varble *et al*. found that the number of viruses that passed between hosts during RD transmission between animals was small, indeed as few as two barcoded viruses initiated infection^25^. Moreover, the nature of the bottleneck was stochastic, in that the only determining factor was the proportion of barcoded genotype in the donor animals. This suggests that the tight bottleneck at RD transmission is not imposed by the requirement to generate within-host variants, but is relevant even for viruses that are already mammalian-adapted.

Here, we also used a genetic tagging approach to monitor the dose of virus transmitted between ferrets by either contact or RD routes. Donor ferrets were infected with a mixed population of two viruses whose genomes differed only by reverse engineered synonymous mutations that could be easily differentiated. We then quantified the mixtures in sentinel animals that acquired infection by the RD route or by DC during co-housing. We found that mixtures were more readily transmitted during contact transmission events, whereas a bottleneck was imposed at RD transmission. Moreover, this was also the case during infections with a mixture of drug-sensitive and drug-resistant variants: drug resistance was only propagated during contact transmission events that allowed the transfer of minority variants, but this occurred even when resistance carried a fitness cost.

## Results

### Engineered influenza viruses allow tracking of genomes during replication and transmission

**Figure 1:**
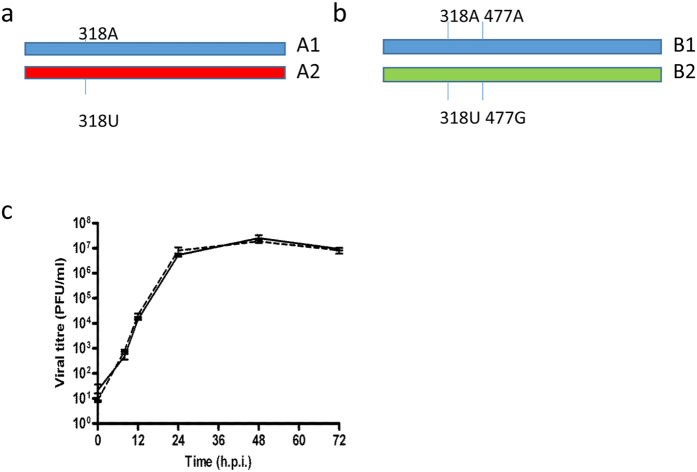
Influenza viruses engineered with synonymous mutations in PB2 gene segment. (**a**) A pair of isogenic viruses based on the prototypic pH1N1 2009 strain A/England/195/2009 were generated using reverse genetics that differed only at nucleotide 318 (A or U) in PB2 (numbering from 5′ end of positive strand) and designated genotype A1 or A2. (**b**) A pair of isogenic viruses were generated using reverse genetics that differed only at nucleotide 318 (A or U) and 477 (A or G) in PB2 and designated genotype B1 or B2. (**c**) Multicycle replication in MDCK cells of viruses that differed only at position 318 and 477 in PB2 gene (B1 and B2). MDCK cells were infected with each virus at low multiplicity (moi = 0.001) and overlaid with serum free DMEM containing 1μg/ml TPCK trypsin. Samples obtained at various times after infection were titrated for infectivity by plaque assay in MDCK cells. With multiple t-tests using the Holm-Sidak method, there are no significant differences between titres of each virus at any time point.

Transmission experiments were performed in ferrets using engineered influenza viruses based on the pandemic H1N1 2009 virus A/England/195/2009 which transmits efficiently between ferrets by both DC and RD routes^2^. In order to trace two different populations of virus replicating in donor animals and transmitting to sentinel ferrets, we employed reverse genetics to generate recombinant viruses that were tagged by introducing synonymous mutations in the PB2 gene (Fig. 1).

We generated a pair of isogenic viruses, that differed by a single nucleotide polymorphism in the PB2 gene at nucleotide position 318 (numbering from 5′ end of positive strand), A vs U (Fig. 1a). A second pair of viruses carried two engineered synonymous PB2 mutations at nucleotides 318 (A vs U) and 477 (A vs G) (Fig. 1b).

The synonymous mutations did not affect virus replication *in vitro*. In a multicycle replication assay in MDCK cells, there was no significant difference in viral titres reached at any time point following infection with either wild type A/Eng/195/2009 virus or the double-tagged virus (Fig. 1c).

### Respiratory droplet transmission imposed a genetic bottleneck

To study the virus population *in vivo*, four separately housed ferrets were directly inoculated with a mixture of viruses differing at nucleotide 318 (A or U) at a 90%:10% infectivity ratio. The virus mix had a total infectivity of 10^4^ plaque forming units (PFU). The following day each infected animal was paired with an RD sentinel ferret introduced into an adjacent cage. Sentinels were exposed to air from their paired infected donors for 10 days as previously described^2^,^26^,^27^. All animals were nasal washed daily.

**Figure 2:**
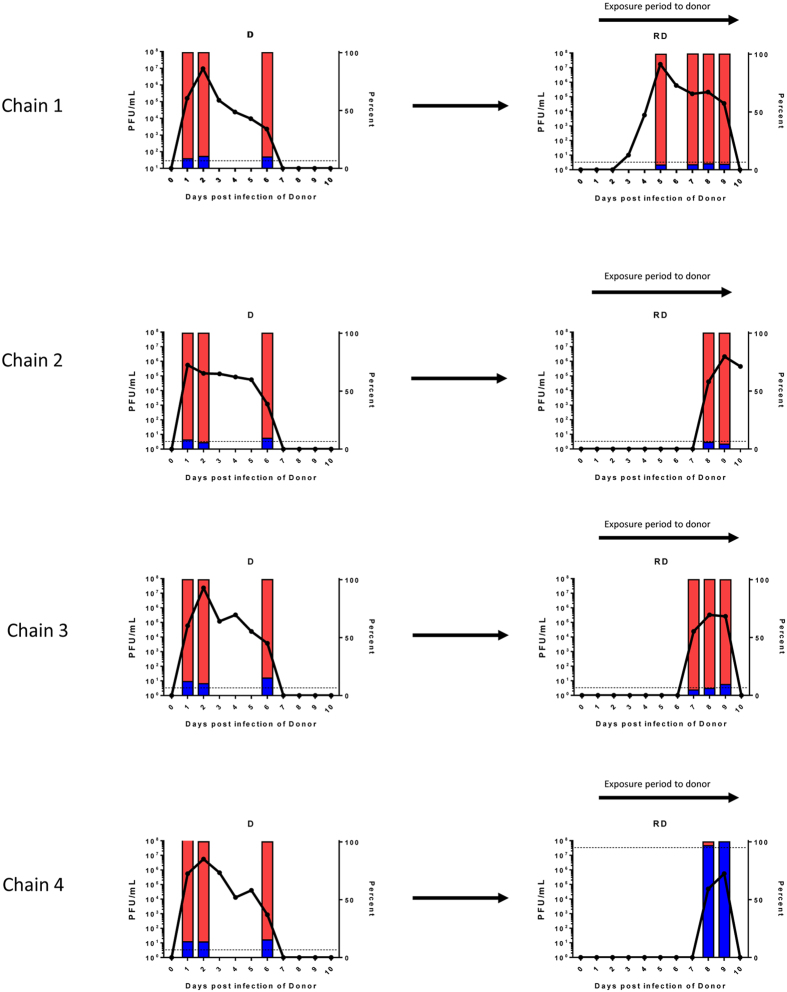
Respiratory droplet transmission of influenza virus between ferrets. Four donor ferrets were infected with a total of 10^4^ PFU of a mixture of two viruses that differed by a single synonymous change at nucleotide 318 (A or U) in PB2 gene in the ratio 90% genotype A1:10% genotype A2. Four RD sentinel animals were exposed day 1 to day 12 by housing in cages adjacent to donor ferrets. Infectivity in nasal washes obtained on each day after infection of donor animals was titrated by plaque assay in MDCK cells and is plotted on left hand side y axis. Percent genotype A1 (red bars) or A2 (blue bars) was established by pyrosequencing RNA extracted from nasal wash and is plotted on right hand side y axis. The dotted lines indicate the limit of detection of pure genotype for the pyrosequencing assay, established as the mean minus 1.96 standard deviation of the mean of four independent measurements of pure genotype A1 or A2 virus.

All four directly inoculated animals shed robust titres of infectious virus from the nose beginning on day 1 and cleared virus by day 7. RD transmission occurred in all four pairs, although the day at which virus was first detected in sentinel animals varied from day 4 to day 8 post exposure (Fig. 2).

The proportion of each viral genotype in nasal washes from directly inoculated animals was quantified by pyrosequencing virus samples collected on days 1 and 2, when animals are most contagious^2^ and on day 6 after several days of infection. Pyrosequencing was also used to quantify the proportion of virus genotypes shed from sentinel recipient animals on the first or second day that infectious virus was detected in the nasal wash and each day thereafter (precluding day 6 of RD1 due to loss of sample material) (Fig. 2). The pyrosequencing assay threshold was 7%, a minority genotype at less than that level could not be differentiated from a pure population (see methods). On day 1 after infection, all four donor animals shed virus with the ratio of genotypes similar to the input inoculated ratio (90%:10%). Over the course of infection the proportion of genotypes shed in nasal wash by any donor animal did not differ more than 3.6% from the proportion measured on day 1. Thus there was no evidence of an *in vivo* fitness cost or gain conferred by the PB2 A318U synonymous mutation and both A1 and A2 virus genotypes replicated robustly.

In contrast, the proportions of virus genotypes shed from the four recipient ferrets who acquired their infection through RD exposure varied considerably (Fig. 2, right hand side). Pyrosequencing indicated that two of the four sentinel animals (RD sentinel, chain 1 and RD sentinel, chain 2), were infected solely by virus bearing the majority A1 genotype. The third animal (RD sentinel, chain 3) was virus-positive for 3 days, on days 7, 8 and 9. Only genotype A1 was detected on day 7 and 8 samples but in the day 9 sample a small but measurable amount of genotype A2 virus was detected (8.4%). The fourth RD exposed ferret (RD sentinel, chain 4) shed virus on day 8 and 9 that was uniquely the A2 genotype, that had been the minority genotype (12.4% to 13.5%) detected in the corresponding donor ferret.

To further confirm the proportion of virus genotypes from each recipient ferret, we pyrosequenced 40 individual plaques from nasal washes collected on the day of peak virus shedding, (or the day after for sentinel 3 due to availability of samples). All 40 plaques from RD sentinels in chain 1 and 2 were of the majority (A1) genotype and all the plaques from RD sentinel in chain were of the minority genotype (A2). Of the 40 plaques from RD sentinel in chain 3, 4 (10%) contained minority (A2) genotype and the remaining 36 (90%) were majority (A1) genotype (Table 1). These data confirmed the previous pyrosequencing results and showed that two animals were infected with only the majority (A1) genotype, one animal with the minority (A2) genotype and one animal with a mixture of the two virus genotypes.

### During contact transmission between ferrets, mixtures of virus genotypes are transmitted

In a second experiment we introduced an additional synonymous nucleotide change to an adjacent region of PB2 (Fig. 1b).

**Figure 3:**
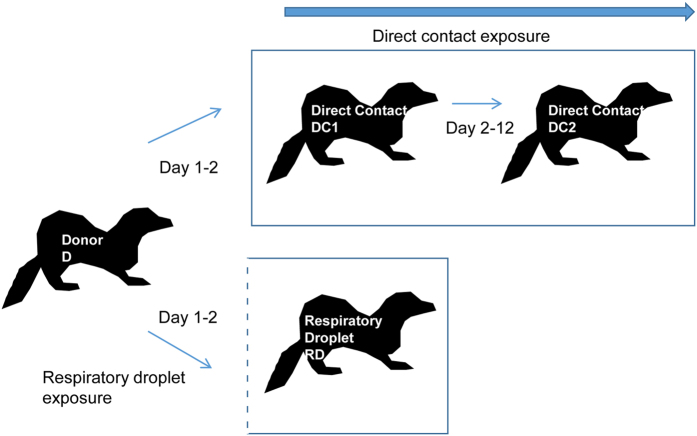
Two chains of transmission of influenza virus by respiratory droplet or direct contact routes. Two donor ferrets one for chain 1 and one for chain 2, were infected with a total of 10^4^ PFU of a mixture of viruses based on influenza A/England/195/2009 that differed only at two synonymous changes in PB2 gene at position 318 (A or U) and 477 (A or G) (B1 and B2). On day 1 post infection of donor, direct contact ferrets (DC1) were co-housed with donor animals and RD exposed ferrets (RD) were housed in adjacent cages that shared air with D and DC1. On day 2 post infection of donor, donor ferrets (D) were removed to separate cages and fresh contact ferrets (DC2) were introduced to the same cage as DC1.

**Figure 4:**
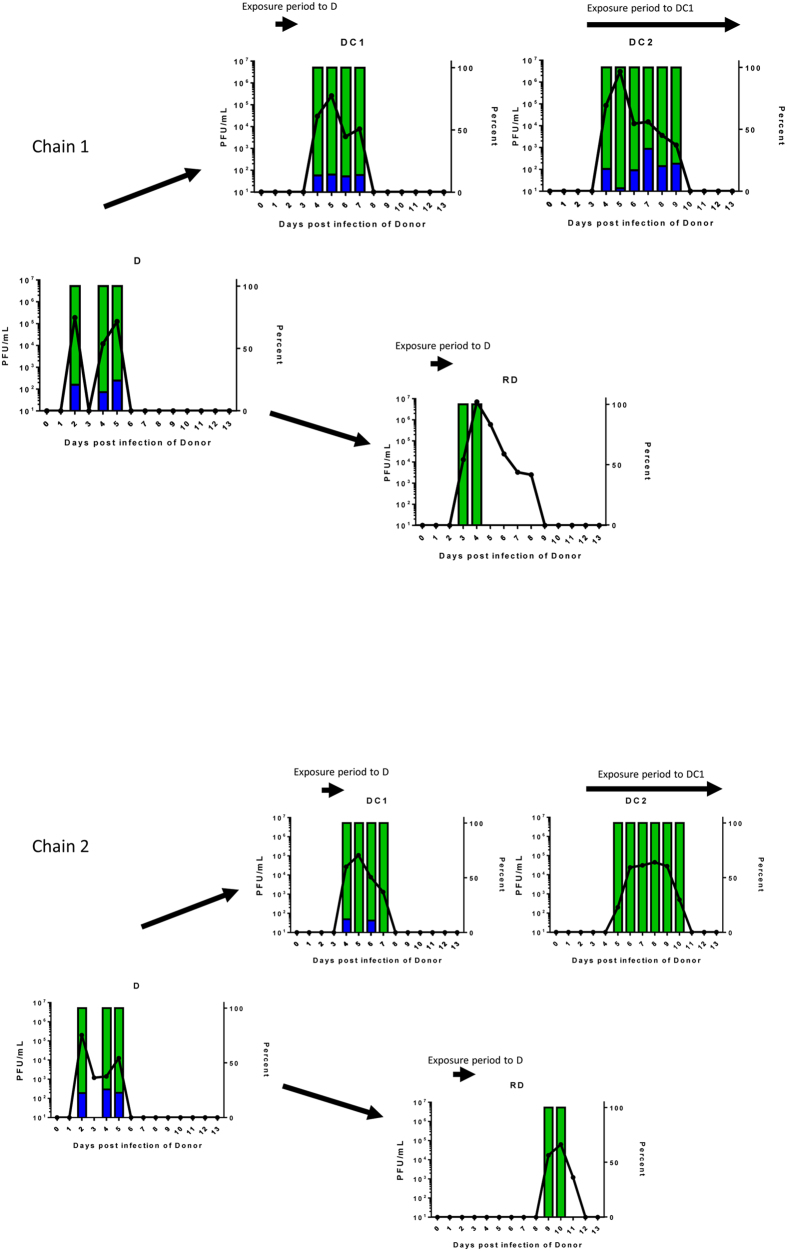
Virus shedding and proportion of genotypes in nasal wash from ferrets infected with a mixture of genotypes with transmission by direct contact or respiratory droplet routes. Infectious virus shed in nasal wash obtained from ferrets after infection or exposure to infected ferrets is plotted as PFU/ml on the right hand side y axis. The percentage of genotype B1 (green) or B2 (blue) established by pyrosequencing RNA extracted from nasal wash is plotted on the left hand side y axis.

Two separate transmission chains involving both contact and RD exposure were set up as illustrated in Fig. 3. In an attempt to further define a likely transmitted dose during the stringent RD transmission event, we increased the proportion of the rarer genotype. For each of the two chains, a ‘donor’ animal (D) was infected with 10^4^ PFU of the 70%:30% mixture of A/England/195/09 virus genotypes containing silent tags at nucleotides 318 and 477 in the PB2 gene. The next day a contact sentinel animal (DC1) was introduced into the same cage and an RD sentinel animal (RD) was placed in the adjacent cage. Exposure of both ferrets DC1 and RD to the donor animal was limited to 24 hours (day 1–2 after donor infection) since we have previously shown that transmission occurs efficiently at this time^2^. On day 2, the donor animal was removed from the cage and exposed sentinel DC1 was housed for a further 10 days with a second naïve contact animal (DC2). The whole transmission chain was performed in duplicate giving rise to chain 1 and chain 2. As before, animals were nasal washed daily and monitored for shedding of infectious virus by plaque assay. Transmission occurred to all animals in the experiment as determined by shedding of infectious virus in nasal wash, although the time between first exposure and virus shedding ranged from 2 to 9 days (Fig. 4, solid lines).

Pyrosequencing results for nucleotide position 477 are shown in Fig. 4 by the histogram bar sizes. The pattern of mixtures analysed by pyrosequencing at nucleotide 318 was the same (correlation coefficient 0.984, p < 0.0001 Figure S1), although controls indicated a slightly higher misreading rate for the 318 assay (data not shown). Thus, the mixtures detected reflected the transmitted genome composition and were not skewed by random mutations that effected loss of the tag.

The two directly inoculated donor animals shed virus at a ratio of approximately 80%:20% genotypes that did not vary over the course of infection. This demonstrated that the genetic tags at positions 318 and 477 did not affect virus fitness *in vivo* (Fig. 4, left hand graphs, D). The difference in genotype ratio from the input (70:30) might be explained by a slightly different ratio of infectious to non-infectious particles in the two virus stocks. In both transmission chains, ferrets who acquired their infection by RD exposure shed uniquely one genotype of virus, which in both cases was the majority species in the donor animals (Fig. 4, bottom graphs, RD).

In contrast, ferrets exposed by the DC route acquired a mixture of virus genotypes. In chain 1, ferret DC1 shed 13.5% minority genotype virus on the first virus-positive day (Fig. 4 top panel). Further along DC transmission chain 1, ferret DC2 also shed a mixture of genotypes, containing between 18% and 22% minority genotype on every day until virus was cleared.

In the other chain of transmission, chain 2, (Fig. 4 lower panels), the first DC exposed animal shed either a mixture of genotypes or pure genotype virus on alternate days. The co-housed contact of this animal, DC2, shed only one virus genotype (the majority type shed from DC1) on any day after contracting infection.

In addition to using pyrosequencing to quantify the proportions of engineered genotypes within each ferret, we also undertook NGS of the whole genomes of viruses in the transmission chains (Tables 2 and 3). These data confirmed that donor ferrets were infected with a mixed population of genotypes that varied at the tagged PB2 nucleotides 318 and 477 and shed these genotypes at a similar ratio as detected by pyrosequencing (for example 78%:22% at position 477 for D1 on day 1). Both RD exposed recipient ferrets were confirmed to be only infected by one (the majority) of these genotypes. In the first transmission chain, NGS also detected a unique pair of genetic variants in the HA gene (nonsynonymous K119M) and M gene (synonymous A570G) present at 100% in virus shed from the RD exposed ferret. The HA and M gene mutations were not detected in the corresponding donor ferret, nor were they present in the second transmission chain, so were clearly not required for RD transmission. A novel nonsynonymous variant in the NS2 (NEP) gene I113T was detected in both samples of infectious virus shed by the RD ferret in the second chain. In this case, the variant nucleotide was present as a mixture with the parental sequence, and as for the HA and M variants above, was not visible in the inoculum or in the virus shed from the corresponding donor animal by NGS.

For the DC transmission events, NGS data at the 1% reporting quality threshold confirmed that mixtures of virus genotypes were propagated by this route. In transmission chain 1, the only variants detected were the engineered tags in PB2 at positions 318 and 477 and mixtures with minority percentages of 20–30% were present in virus from both ferrets DC1 and DC2. In the second contact transmission chain, the mixture of engineered tags was propagated to the first DC ferret. Additionally, one mutation in the PB2 gene segment (nonsynonymous E118G) plus two mutations in the PA gene segment (one nonsynonymous A404T and one synonymous C1449T) were detected in the first DC animal that were not detected in the donor. Upon further transmission to ferret DC2, the engineered tags at position 318 and 477 in PB2 were not transmitted as a mixture, (as had been shown by pyrosequencing), but a mixed genotype at E118G in PB2 and A404T PA was maintained.

Taken together these data suggest that RD transmission between ferrets exerts a tight bottleneck in which mixed genotypes tend to be purified, whereas DC transmission events can propagate genetic mixtures.

### Estimation of the transmitted dose of influenza virus in respiratory droplet transmission between ferrets

**Figure 5:**
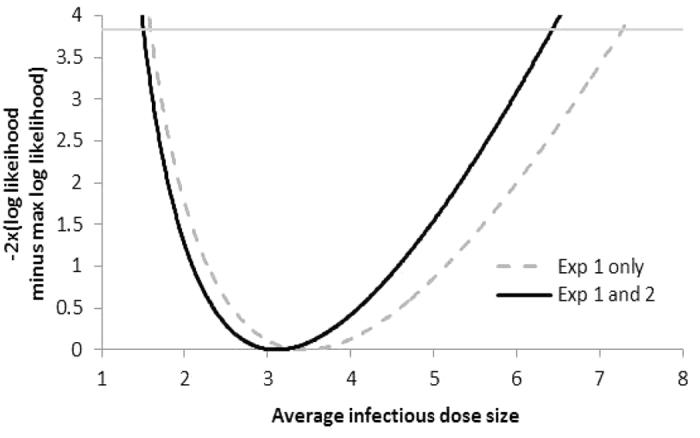
Estimated average dose size for respiratory droplet transmission between ferrets. Individual infectious doses sizes were assumed to be Poisson distributed, and not be less than one (see material and methods for the statistical analysis). Estimate of the average dose size using data from experiment 1 (4 donor/RD sentinel pairs) and 2 (2 donor/RD sentinel pairs) shown in solid black line. Estimate using data only from experiment 1 shown in grey.

The pyrosequencing and NGS data were used to estimate the average infectious dose size during RD transmission, assuming individual infectious dose sizes were Poisson distributed, and that infection in the recipient animal could not be initiated with less than a single infectious virus particle (see methods for the statistical analysis). The average infectious dose size for RD transmission, (μ/(1 − e^−μ^)), was estimated to be 2.83 infectious genomes (95% confidence interval 1.44–5.74) based on both experiments 1 and 2 and to be 3.45 infectious genomes (95% confidence interval 1.59–7.28) based only on experiment 1 (Fig. 5).

For transmission that occurred between ferrets in direct contact, the dose size was clearly higher. Using the same approach as described above, and assuming a Poisson distribution of dose sizes, the estimated average dose size distribution was 28.2 infectious genomes. The lower limit was 3.7. It was not possible to put an upper limit on this value.

### Propagation of oseltamivir-resistant pH1N1 virus from mixtures by direct contact transmission

One important scenario in which the propagation of mixtures of virus of different genotypes might carry clinical significance is during antiviral treatment. Here the evolution of drug-resistant mutants can occur and conditions in which mixtures of genotypes are transmitted might facilitate onwards transmission of resistance to other patients, in a nosocomial setting for example.

**Figure 6:**
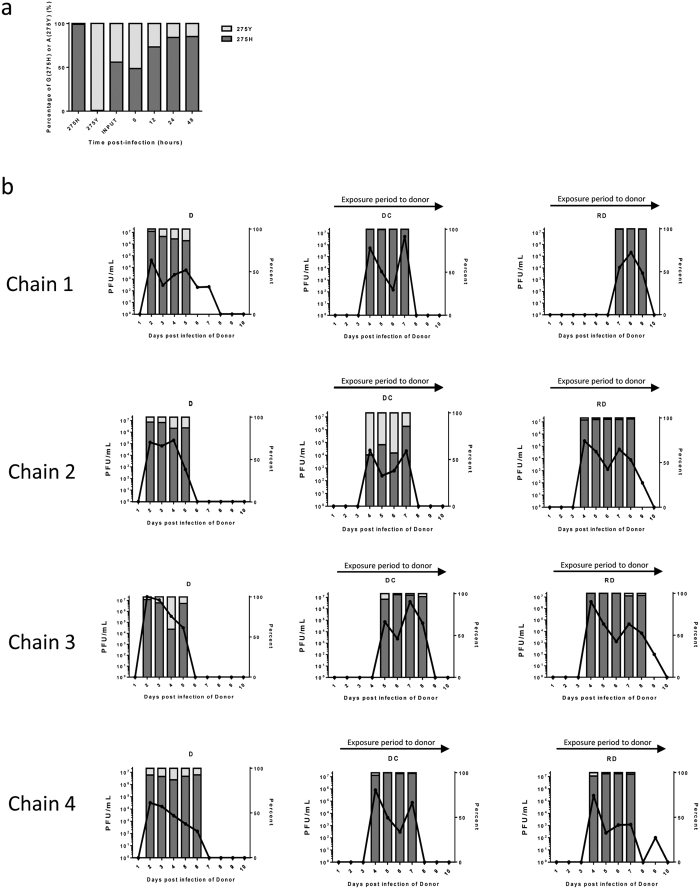
Competitive replication of oseltamivir-resistant and oseltamivir-sensitive pH1N1 virus in human airway epithelial cells and transmission between ferrets. (**a**) Human airway epithelial cells were infected with a mix of the oseltamivir-sensitive and resistant viruses (50%:50%). The percentage of genotype 275H (dark grey bars) or 275Y (light grey bars) was established by pyrosequencing RNA extracted from virus released at the apical side of the cultures. (**b**) Four donor ferrets were infected with a total of 10^4^ PFU of a mixture of oseltamivir-sensitive and -resistant viruses. On day 1 post infection of donor, direct contact ferrets (DC) were co-housed with donor ferrets and respiratory droplet ferrets (RD) were housed in adjacent cages. Percentage genotype 275H (dark grey bars) or 275Y (light grey bars) was established by pyrosequencing RNA extracted from nasal wash.

To model this scenario, we generated a pair of recombinant viruses based on a third wave pH1N1 virus, A/England/687/2010, that differed in the neuraminidase protein only by the amino acid H275Y, a well characterized oseltamivir-resistance-conferring mutation^28^,^29^,^30^. In five separate wells of primary human airway epithelial cells co-infected with an equal mixture of the two viruses, the sensitive virus genotype predominated after several rounds of multicycle replication (Fig. 6a) suggesting that, in this virus genetic background, 275Y carried a small but measurable fitness cost for replication in the human airway.

Four donor ferrets were directly inoculated with 10^4^ PFU of a mixture of 70% oseltamivir-sensitive and 30% resistant viruses, and naïve sentinel animals were exposed from day 1 by the DC or RD route to each donor animal. All animals shed virus from the nose for between 3 and 7 days (Fig. 6b). Pyrosequencing to detect proportions of genotypes H275 or Y275 illustrated that all four RD transmission events led to loss of the oseltamivir-resistant genotype 275Y and three of the four contact transmission events also showed this pattern. However, in the fourth direct contact transmission event (chain 2), the recipient animal shed a mixture of virus genotypes in which the resistant genotype was maintained over the entire infection course.

## Discussion

Influenza infections can be initiated by inoculation of very low doses of virus^6^,^31^,^32^,^33^,^34^,^35^,^36^,^37^. Some studies suggest infectious doses to be as low as single infectious units.

However, the fact that a low dose of virus *can* initiate infection when directly inoculated into animals or humans does not necessarily mean that during a transmission event, only a low dose is passed on. Here, we used a genetic approach to address this question by engineering recombinant influenza viruses that could be distinguished by a unique nucleotide tag. This approach has been previously used to study bottlenecks in both viral and bacterial systems (reviewed in Abel *et al*.^38^). In some of the previously reported studies, complex mixtures containing hundreds of different tags have been employed^39^. However, it appears that, during influenza transmission, the bottleneck size is small enough that the analysis was possible using a simple mixture of two distinguishable genotypes. In this way we were able to conclude that, during RD transmission of pH1N1 2009 influenza viruses between ferrets, the transmitted dose was on average 3 infectious virions. That the RD-transmitted dose is low may be further supported by analysing the kinetics of virus shedding in RD recipient animals. Some of the animals who received their transmitted virus by the RD route had a delayed time to first virus and lower peak shedding compared to donor animals or DC recipients (for example chain 2 Fig. 4 and chain 1 Fig. 6), and this slower kinetic pattern is reminiscent of observations on shedding from animals directly inoculated with lower doses of influenza^31^,^40^,^41^. It may be that such analyses of virus kinetics could offer an alternative strategy to estimate the transmitted dose but our study was not designed or powered to directly test this.

During DC transmission events between co-housed animals the estimated transmitted dose was higher, by 10 fold. Increasing the numbers of sequence tags would allow a more accurate estimation of the DC transmitted dose. Nonetheless the data suggest that animals in close contact exchange higher virus doses, or that they are susceptible to repeated superinfections during the exposure period. Indeed, it is interesting to note that virus shedding occurred over a longer time period from animals who acquired their infections by direct contact during continuous exposure (DC2 animals in Fig. 4 vs DC1 animals in the same transmission chains), This may be due to repeated transmission events topping up the viral load in an infected recipient, although in chain 2, the absence of tagged virus in animal DC2 despite exposure to virus with tag shed from DC1 on days 5 and 7 might argue against this. Our estimates of transmitted dose align well with those from Varble *et al*.^25^ and McCaw *et al*.^37^ who both estimated that only small numbers (2 or 3) of influenza viruses are transmitted between ferrets. Taken together, these data may explain why some non-adapted animal influenza viruses might transmit by DC but not by the RD route; as a dose greater than the minimal infectious dose is more likely to be passed on during co-housing, benefiting viruses that require a relatively high ID_50_.

Varble *et al*.^25^ had suggested that the transfer of virus by the RD route was stochastic, whereas using an NGS approach, Wilker *et al*.^22^ had seen that for H5N1 RD transmission, minority variants were transmitted from the virus pool in the donor animals, leading them to suggest the stringent RD bottleneck was at least partly accounted for by a requirement for a more transmissible genotype. In the present study, we detected a set of novel mutations in HA and M in one RD recipient animal (chain 1 Table 2). When the threshold for reporting of variants for the NGS sequencing was lowered from 1% to 0.01%, the variant bases were detected in the donor, but in less than 0.2% of the reported reads. We cannot exclude that these variants in the donor samples were due to reverse transcriptase, polymerase or sequencing errors. Moreover, these mutations were not present in the RD recipient in the second chain suggesting they were not on their own required for transmission.

The mechanism underlying the tight bottleneck for RD transmission is as yet unknown. One reason for the airborne transmitted dose being so low is likely to be the attrition of infectivity whilst virions are outside a host in respiratory droplets, both by dilution and inactivation in the environment, and the loss of virus present in large and medium size droplets (diameter >5um) that drop out of the air under Stokes Law before reaching the adjacent cage. Only a proportion of virus is shed from the infectious animal in droplets small enough to remain airborne beyond the distance separating the ferret cages^34^,^32^,^42^. On the other hand when transmission occurs over smaller distances, more virus expelled in larger droplets (>5 um) can be transferred. It may also be that several virus particles can be carried within a single large droplet, making simultaneous infection by several genomes possible. Other non-exclusive explanations might be a restriction on superinfection by competing viruses, either directly or by triggering host innate defences^38^,^43^. The case where only minority variant was transmitted, (RD in chain 4, Fig. 2), suggests that a simple stochastic model may be insufficient to explain this event and that some level of viral interference may play a role in determining the bottleneck.

A narrow transmission bottleneck has also been reported during natural transmission of HIV, Hepatitis C virus, and Venezuelan equine encephalitis virus, although these viruses are transmitted by different routes than influenza virus^41^,^44^,^45^. On the other hand, during natural or experimental transmission of influenza virus in two domesticated mammalian hosts, horses and pigs, some genome diversity was transmitted^46^,^47^,^48^. In both of these large domestic animal hosts, extensive close contact between donor and recipient takes place giving opportunity for transfer of higher doses at the time of virus transfer. More recently, a study of influenza virus diversity during household transmissions suggested dose sizes of 100–200 viral particles, and this may imply that most of these transmission events followed direct contact between donor and recipient^49^.

The contribution of different transmission routes in human influenza outbreaks is difficult to quantify and is poorly understood. It is likely that several different routes including respiratory droplets and aerosols as well as close contact and fomites all play a role^3^. To understand the consequences of different routes of transmission on viral evolution, we studied transmission from animals inoculated with mixtures of antiviral drug-sensitive and resistant viruses. In the absence of antiviral administration, the resistance phenotype engineered into a third wave UK strains of pH1N1 virus carried a small fitness cost since it was outcompeted by drug sensitive virus during replication *in vitro* in primary human airway cells. Butler *et al*. had previously shown that late wave Australian strains of pH1N1 2009 drug-resistant virus could be transmitted to DC-exposed ferrets even in the presence of drug sensitive virus^50^. They did not include the RD exposure route in their protocol. In our study, drug-resistant virus was not transmitted to RD recipient animals, which might be explained by a stringent RD transmission bottleneck exerting a purifying selection for the fitter drug-sensitive virus. However, drug-resistant virus was detected in 1of 4 DC recipient animals, illustrating that close-contact or fomite transmission, by allowing transfer of larger virus doses, is one way in which ongoing evolution of influenza viruses could be propagated.

## Methods

### Cells and virus

MDCK cells were maintained in Dulbecco’s modified Eagle’s medium (DMEM; Gibco, Invitrogen) supplemented with 10% FBS and 1% penicillin/streptomycin (Sigma-Aldrich).

A/England/195/2009 was a prototypic first wave isolate of the 2009 pH1N1 pandemic from the UK. A/England/687/2010 was a typical third wave isolate of pH1N1 virus from the UK. Reverse genetics systems by which recombinant viruses can be rescued and genetically manipulated were previously described^2^. Briefly virus was rescued following 12 plasmid transfection of 293-T cells and co-culture with MDCK cells. To rescue virus altered in PB2 segment site directed mutagenesis was performed with a Stratagene Quick change kit on the pol I PB2 plasmid and mutated plasmid used in place of wild type in the virus recue.

### Plaque assay

Plaque assays were performed using MDCK cells as previously described^31^. Briefly, 100% confluent cell monolayers were inoculated with 100 μl of serially diluted samples and overlaid with 0.6% agarose (Oxoid) in supplemented DMEM with 2 μg trypsin (Worthington) ml^−1^ and incubated at 37 °C for 3 days.

### Pyrosequencing

PCR and sequencing primers for pyrosequencing assays were designed using the Qiagen PyroMark assay design software, against the A/England/195/2009 PB2 sequence (Accession GQ166656). Primer sequences are; PCR forward: 5′ Biotin labelled-GCCGTAACATGGTGGAATAGGAAT; PCR Reverse: TCTTGCCCCCACTTCATTTG; A318U sequencing: TTAGGGTAATGAACTGTACT; A477G sequencing: TTCCATAATCACATCCTG. The assays were designed such that both target single nucleotide polymorphisms were contained in the same PCR amplicon, to eliminate errors due to differential amplification (One step RT-PCR cycling conditions: 50 °C: 30 min; 95 °C:15 min; (40 cycles of 94 °C: 0.5 min; 61 °C: 0.5 min; 72 °C: 1 min) 72 °C: 10 min). Pyrosequencing reactions were performed according to manufacturers’ instructions (20 μl PCR product; sequencing primer at final concentration of 0.44 μM; annealing at 80 °C for 2 minutes).

To determine the limit of detection of the target nucleotide polymorphism, i.e. the frequency with which it miscalled U for A at nucleotide 318, four independent assays were performed from a pure (100%) stock of the genotype A1 virus (PB2 318A). This gave a mean value of 95.1% A1 virus with a standard deviation of 1.2%. We defined the limit of detection of pure genotype A1 as 92.7% (the mean minus 1.96 standard deviations). Thus any sample with a % genotype A2 less than 7.3% (indicated by the dotted line on the graphs in Fig. 2) was deemed to contain ‘pure’ genotype A1. Similarly the threshold for deeming a sample to contain only genotype A2 was determined as 95.5% (mean minus 1.96 standard deviations of four independent measurements of pure genotype A2). This threshold is indicated by the dotted line for RD sentinel in chain 4 in Fig. 2.

The 477 pyrosequencing assay did not give any error; stocks of pure B1 virus recorded 100% A nucleotide and B2 virus stocks measured 0% A.

### Animal Studies

Female ferrets (20–24 weeks old) weighing 750–1000 g were used. After acclimatization, sera were obtained and tested by virus neutralization assay for antibodies against A/England/195/2009, pH1N1. All ferrets were seronegative for influenza antibodies at the start of the experiments. Body weight was measured daily, and strict procedures were followed to prevent aberrant cross-contamination between animals. Sentinel animals were handled before inoculated animals, and work surfaces and handlers’ gloves were decontaminated between animals. Inoculated ferrets were lightly anaesthetized with ketamine (22 mg kg^−1^) and xylazine (0.9 mg kg^−1^) and then inoculated intranasally with virus diluted in phosphate buffered saline (PBS) (0.1 ml per nostril). All animals were nasal washed daily, while conscious, by instilling 2 ml PBS into the nostrils, and the expectorate was collected in modified 250 ml centrifuge tubes. The nasal wash expectorate was used for virus titration by plaque assay and RNA was extracted for pyrosequencing. The limit of virus detection in the plaque assays was 10 PFUml^−1^.

### Ethics Statement

All work was approved by the local genetic manipulation (GM) safety committee of Imperial College London, St. Mary’s Campus (centre number GM77), and the Health and Safety Executive of the United Kingdom and carried out in accordance with the approved guidelines. All animal research described in this study was approved and carried out under a United Kingdom Home Office License, PPL 70/7501 in accordance with the approved guidelines.

### Statistical methods

The data were used to estimate the average infectious dose size, assuming individual infectious dose sizes (denoted by X) were Poisson distributed, conditional on 1 being the minimum dose size. This was achieved by calculating, for each potential conditional average infectious dose size (μ/(1 − e^−μ^), the probability of infectious dose size X infectious genomes. Given the infectious dose size X, the probability was calculated of M of the X infectious genomes in the infectious dose being the majority strain (i.e. M majority strain genomes and (X-M) minority strain genomes, so M could vary from 0 to a maximum of X, the infectious dose size), given the donor animal was infected with 90% majority strain and 10% minority strain. For experiment 1, given that M of the X infectious genomes were the majority strain, the probability was calculated of Y of the 40 sampled plaques being the majority strain (i.e. Y majority strain plaques and (40-Y) minority strain plaques, so Y could vary from 0 to a maximum 40). The data likelihood for infected ferret i in experiment 1 (L_1i_) with Y majority strain plaques among the 40 sampled plaques was obtained from the products of these three probabilities such that:


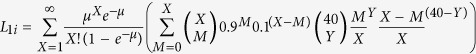


The data likelihood for infected ferret j in experiment 2 (L_2j_) is simplified in form because ferrets will only be infected with purely the majority strain if all X infectious genomes in the infectious dose were the majority strain:


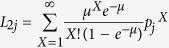


where p_j_ was the average proportion of the majority strain measured in the donor ferret which infected ferret j (for donor ferret in chain 1 this was 77.3% and for donor ferret in chain 2 this was 80.9%). The full data log likelihood as a function of μ, the average infectious dose size, is obtained from ln(∏_i_L_1i_)+ln(∏_j_ L_2j_). Likelihood-ratio-based confidence intervals were obtained from the log likelihood profile for each estimate of μ.

## Additional Information

**How to cite this article**: Frise, R. *et al*. Contact transmission of influenza virus between ferrets imposes a looser bottleneck than respiratory droplet transmission allowing propagation of antiviral resistance. *Sci. Rep.* **6**, 29793; doi: 10.1038/srep29793 (2016).

## Supplementary Material

Supplementary Information

## Figures and Tables

**Table 1:** Results of pyrosequencing 40 individual plaques picked from nasal wash samples from RD exposed ferrets on the days post exposure indicated, the peak day of virus shedding for these animals.

Sentinel	Day post exposure	Proportion of picked plaques with genotype A2
1	5	0/40
2	9	0/40
3	9	4/40
4	9	40/40

**Table 2:** Substitutions in influenza A/England/195/2009 (pH1N1) genomes in nasal wash samples from ferret transmission chain 1 (Fig. 4) as observed by next generation sequencing.

Chain #1							
Ferret	Day Post Infection	SNP	Segment	Genotype		Coverage^a^	AA change^b^
Donor	1	A318T	PB2	A26%	T74%	3887	SYN
		A477G		A22%	G78%	3666	
		A7121T	HA	A100%		4371	
		A11901G	M	A100%		13718	
	2	A318T	PB2	A32%	T68%	1536	
		A477G		A24%	G76%	1563	
		A7121T	HA	A100%		2597	
		A11901G	M	A100%		8737	
	5	A318T	PB2	A31%	T69%	1110	
		A477G		A27%	G73%	875	
		A7121T	HA	A100%		7566	
		A11901G	M	A100%		23140	
DC1	4	A318T	PB2	A16%	T84%	1708	
		A477G		A12%	G88%	1517	
DC2	4	A318T		A27%	T73%	3198	
		A477G		A23%	G77%	3030	
	9	A318T		A30%	T70%	**139**	
		A477G		A32%	G68%	**88**	
RD	3	A318T	PB2	T100%		3364	
		A477G		G100%		3300	
		A7121T	HA	T100%		6511	K136M (K119M)
		A11901G	M	G100%		18928	SYN
	4	A318T	PB2	T100%		**29**	
		A477G		A4%	G96%	**26**	
		A7121T	HA	T100%		**66**	K136M (K119M)
		A11901G	M	G100%		190	SYN

The genotype is expressed as a percentage of the total number of reads with that nucleotide (with a 1% reporting quality threshold). The coverage reflects the number of reads that pass the quality threshold, ^a^values in bold indicate low depth. Where the nucleotide variation does not cause an amino acid change the abbreviation SYN denotes an synonymous change, where there is an non-synonymous change this is denoted by the IUPAC-IUB codes and the amino acid position is listed from the primary methionine, ^b^H1 numbering below in parenthesis for the HA segment.

**Table 3:** Substitutions in influenza A/England/195/2009 (pH1N1) genomes in nasal wash samples from ferret transmission chain 2 (Fig. 4) as observed by next generation sequencing.

Chain #2							
Ferret	Day Post Infection	SNP	Segment	Genotype		Coverage^a^	AA change^b^
Donor	1	A318T	PB2	A30%	T70%	2441	SYN
		A477G		A25%	G75%	1883	
		A7121T	HA	A100%		4561	
		A11901G	M	A100%		14626	
		T13126C	NS2	T100%		1443	
		G5770A	PA	G100%		2449	
		C6009T	C100%		3482		
	2	A318T	PB2	A28%	T72%	3197	
		A477G		A23%	G77%	2735	
		G5770A	PA	G98.5%	A1.5%	3559	A404T
		C6009T		C100%		4992	SYN
		A7121T	HA	A100%		4685	
		A11901G	M	A100%		18743	
		T13126C	NS2	T100%		2443	
DC1	4	A318T	PB2	A25%	T75%	4417	
		A477G		A20%	G80%	3787	
		A563G	A92%	G8%	3791	E188G	
		G5770A	PA	G90%	A10%	2449	A404T
		C6009T		C88%	T12%	6291	SYN
	7	A318T	PB2	A12%	T88%	626	
		A477G		A10%	G90%	540	
		A563G	A82%	G18%	558	E188G	
		G5770A	PA	G71%	A29%	1553	A404T
		C6009T		C94%	T6%	1876	SYN
DC2	5	A318T	PB2	A2%	T98%	281	
		A477G		G100%		301	
		A563G	A90%	G10%	282	E188G	
		G5770A	PA	G83%	A17%	407	A404T
		C6009T		C89%	T11%	595	SYN
	10	A318T	PB2	T100%		**11**	
		A477G		G100%		**8**	
		A563G	A88%	G12%	**8**	E188G	
		G5770A	PA	G85%	A15%	**26**	A404T
		C6009T		C68%	T32%	**53**	SYN
RD	9	A318T	PB2	T100%		2729	
		A477G		G100%		3261	
		A7121T	HA	A100%		5243	
		A11901G	M	A100%		15243	
		T13126C	NS2	T24%	C76%	3657	I113T
	10	A318T	PB2	T100%		642	SYN
		A477G		G100%		651	
		A7121T	HA	A100%		2945	
		A11901G	M	A100%		24350	
		T13126C	NS2	T5%	C95%	4191	I113T

The genotype is expressed as a percentage of the total number of reads with that nucleotide (with a 1% reporting quality threshold). The coverage reflects the number of reads that pass the quality threshold, ^a^values in bold indicate low depth. Where the nucleotide variation does not cause an amino acid change the abbreviation SYN denotes an synonymous change, where there is an non-synonymous change this is denoted by the IUPAC-IUB codes and the amino acid position is listed from the primary methionine, ^b^H1 numbering below in parenthesis for the HA segment.
