# Supplementary material for: Contact transmission of influenza virus between ferrets imposes a looser bottleneck than respiratory droplet transmission allowing propagation of antiviral resistance
Source: Sci Rep. 2016 Jul 19;6:29793. doi: 10.1038/srep29793 (PMC4949428; doi:10.1038/srep29793)
Supplement: Supplementary Information [file srep29793-s1.pdf]

Title: Contact transmission of influenza virus between ferrets imposes a looser bottleneck than respiratory droplet transmission allowing propagation of antiviral resistance

Authors: Rebecca Frise[1], Konrad Bradley[1], Neeltje van Doremalen[1,4], Monica Galiano[2], Ruth A. Elderfield[1], Peter Stilwell[1,5], Jonathan W. Ashcroft[1,6], Mirian Fernandez-Alonso[2,7], Shahjahan Miah[2], Angie Lackenby[2], Kim L. Roberts[1,8], Christl A. Donnelly[3], and Wendy S. Barclay[1,*].

Supplementary Figure S1

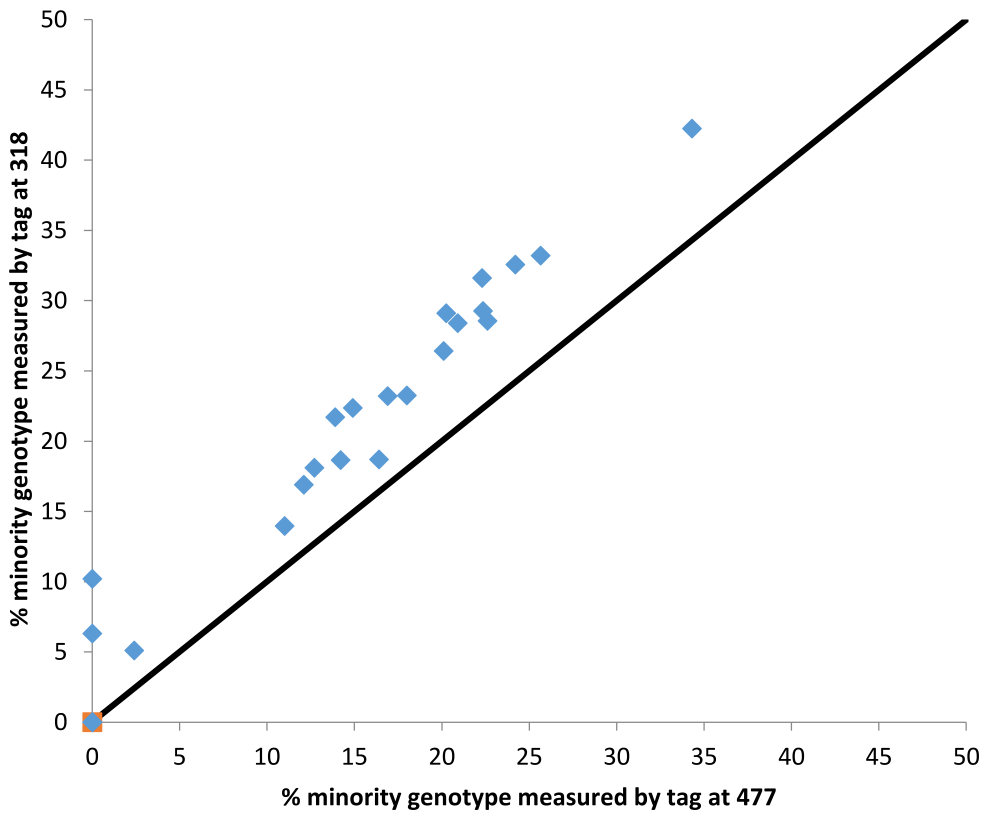

Correlation between measurement of proportions of virus genotype using either of the two engineered tags in PB2 segment.

Data for 38 separate nasal washes measured using pyrosequencing for either tag at nucleotide 318 or tag at nucleotide 477 were plotted. The solid line shows a hypothetical equality.
